# The PI3K/Akt/mTOR Pathway Is Implicated in the Premature Senescence of Primary Human Endothelial Cells Exposed to Chronic Radiation

**DOI:** 10.1371/journal.pone.0070024

**Published:** 2013-08-01

**Authors:** Ramesh Yentrapalli, Omid Azimzadeh, Arundhathi Sriharshan, Katharina Malinowsky, Juliane Merl, Andrzej Wojcik, Mats Harms-Ringdahl, Michael J. Atkinson, Karl-Friedrich Becker, Siamak Haghdoost, Soile Tapio

**Affiliations:** 1 Helmholtz Zentrum München, German Research Center for Environmental Health, Institute of Radiation Biology, Neuherberg, Germany; 2 Centre for Radiation Protection Research, Department of Molecular Biosciences, The Wenner-Gren Institute, Stockholm University, Stockholm, Sweden; 3 Institute of Pathology, Technische Universität München, Munich, Germany; 4 Research Unit Protein Science, Helmholtz Zentrum Muenchen, German Research Center for Environmental Health (GmbH), Neuherberg, Germany; 5 Department of Radiation Oncology, Klinikum Rechts der Isar, Technische Universität München, Munich, Germany; Northwestern University Feinberg School of Medicine, United States of America

## Abstract

The etiology of radiation-induced cardiovascular disease (CVD) after chronic exposure to low doses of ionizing radiation is only marginally understood. We have previously shown that a chronic low-dose rate exposure (4.1 mGy/h) causes human umbilical vein endothelial cells (HUVECs) to prematurely senesce. We now show that a dose rate of 2.4 mGy/h is also able to trigger premature senescence in HUVECs, primarily indicated by a loss of growth potential and the appearance of the senescence-associated markers ß-galactosidase (SA-ß-gal) and p21. In contrast, a lower dose rate of 1.4 mGy/h was not sufficient to inhibit cellular growth or increase SA-ß-gal-staining despite an increased expression of p21. We used reverse phase protein arrays and triplex Isotope Coded Protein Labeling with LC-ESI-MS/MS to study the proteomic changes associated with chronic radiation-induced senescence. Both technologies identified inactivation of the PI3K/Akt/mTOR pathway accompanying premature senescence. In addition, expression of proteins involved in cytoskeletal structure and EIF2 signaling was reduced. Age-related diseases such as CVD have been previously associated with increased endothelial cell senescence. We postulate that a similar endothelial aging may contribute to the increased rate of CVD seen in populations chronically exposed to low-dose-rate radiation.

## Introduction

Cardiovascular disease (CVD) – pathologies of the heart, blood vessels and the vascular system of the brain– is the leading cause of morbidity and mortality in the Western world [1] and accounts for nearly one-third of deaths worldwide [2]. Populations chronically exposed to low doses of ionizing radiation, either occupationally or due to environmental contamination, show an increased risk of cardiovascular disorders. Thus, an increased risk for ischemic heart disease was found amongst radiation workers in the Chernobyl liquidator cohort [3]. In the cohort of Mayak nuclear facility workers much of the evidence for an increased risk for ischemic heart disease arose from workers with cumulative external doses greater than 1 Gy, although the data are consistent with a linear dose response curve. In this cohort, the individual external gamma-ray doses ranged from below 100 mGy to more than 5 Gy [4]. Employees in the nuclear industry at British Nuclear Fuels and Canadian nuclear workers showed an increasing trend for circulatory disease mortality associated with the radiation dose [5], [6]. Similarly, a study amongst the U.S. nuclear power industry workers indicated a strong positive and statistically significant association between radiation dose and mortality from atherosclerotic heart disease, the mean dose being as low as 30 mGy [7]. Taken together, the recent epidemiological data indicate a residual risk at lower cumulative doses and dose rates than previously estimated, challenging radiation protection authorities to provide more precise risk estimates for CVD at chronic exposures.

A large group of people receive repetitive low-dose exposures from medical radiation for imaging purposes [8]. Especially X-ray computed tomography (CT) scanning is a widely used radiodiagnostic method with absorbed tissue doses around 10 to 100 mGy from single CT examinations [9]. Consequently, repeated CT examinations during follow-up procedures lead to an even higher cumulative radiation exposure. Although only around 11% of all imaging procedures in the diagnostic sector are CT scans, they represent around 70% of the total radiation exposure from medical imaging [10]. Although the immediate benefit for the patient often outweighs the possible long-term consequences as in the case of cardiac CT scans [11], the pros and cons should be balanced in each case to exclude their overuse.

The vascular endothelium is a strong target candidate for radiation due to its intrinsic high level of sensitivity to radiation [12]. Endothelial cells enter cellular senescence, also known as replicative senescence or Hayflick limit, where normal diploid cells cease to divide [13]. Senescent cells remain metabolically active and generally adopt phenotypes including flattened cell morphology, altered gene and protein expression, and positive senescence-associated β-galactosidase (SA-ß-gal) staining [14]. Exposure of endothelial cells to acute high doses of ionizing radiation inhibits proliferation and induces premature senescence *in vitro* [15]. Additionally, the *in vitro* invasion and migration activities of both bovine aortic endothelial cells (BAECs) and human umbilical vein endothelial cells (HUVECs) were repressed after an acute 8 Gy gamma dose if the cells were growing exponentially during irradiation [15]. Treatment of confluent monolayers of BAECs with an acute dose of gamma rays resulted in the appearance of cells with an enlarged surface area that were morphologically similar to senescent cells [16]. The majority of these cells stained positively for the senescence marker SA-ß-gal. The incidence of this senescence-like phenotype increased with dose (5–15 Gy) and time after irradiation.

*In vivo*, senescent endothelial cells have been found in vascular lesions appearing after radiation therapy [17]. High acute doses of ionizing radiation are known cause damage to coronary arteries and the cardiac microvasculature. This leads to a diffuse fibrotic injury to the pericardium and myocardium, with endothelial damage being observed in all these cases [18], [19].

At sites overlying atherosclerotic plaques, an increase in senescent endothelial cells have been found in the human aorta and coronary arteries, indicating that endothelial senescence is associated with normal plaque formation in non-exposed populations [20]. Mitchel et al. investigated the effect of low radiation doses given at low dose rates on the plaque formation [21]. Atherosclerosis was investigated in ApoE-deficient C57BL/6 mice after exposure to doses between 0.025 Gy and 0.5 Gy, given at two dose rates (150 mGy/min and 1 mGy/min) [21]. Considering the plaque size and number, doses given at the lower dose rate during either early- or late-stage atherosclerosis were protective, whereas high-dose-rate exposure given to young animals produced both protective and detrimental effects. This study suggests that low doses may influence plaque formation by more than one mechanism and that dose rate is an important parameter.

A study using a subset of animals from the Janus series of experiments compared acute or fractionated exposures of gamma or neutron radiation on the hazards associated with the development of cancer and non-cancer diseases of the liver, lung, kidney or vascular system [22]. Fractionation played a significant role in both cancer and non-cancer disease related risk in both gamma and neutron irradiated animals; fractionation was significantly associated with a longer survival across multiple non-cancer diseases.

We have shown previously that chronic low-dose-rate (4.1 mGy/h) exposure is able to trigger premature senescence in HUVECs. Our data suggested that chronic radiation-induced DNA damage and oxidative stress activate the p53/p21 pathway [23]. This inhibited the replicative potential of HUVECs and led to their premature senescence [23]. Several senescence-related biological pathways were influenced by the chronic radiation exposure, including cytoskeletal organisation, cell-cell communication and adhesion, and inflammation.

The aim of the present study was to investigate whether continuous radiation exposure using even lower dose rates (1.4 mGy/h and 2.4 mGy/h) than in the previous study could induce premature senescence in HUVECs. The effect of these dose rates on biological pathways associated with normal senescence was investigated using reverse phase protein arrays (RPPA) and Isotope Coded Protein Label (ICPL) technology in combination with tandem mass spectrometry (LC-MS/MS). This study raises the question whether there is a lower limit in the dose rate at which there is no measurable induction of premature senescence.

## Materials and Methods

### Chronic gamma irradiation of HUVECs

Ionizing radiation was performed in a custom-made cell culture incubator modified to hold a ^137^Cs-gamma source exposing cells to dose rates of 1.4 mGy/h and 2.4 mGy/h. Irradiation was carried out continuously except during replacement of culture medium and passaging of cells which lasted from 30 min to 1 hour. Sham irradiated cells (control) were grown in an identical incubator but without exposure to radiation.

### Cell culture conditions and cell growth kinetics

HUVECs (Invitrogen, Paisley, UK) were obtained from a single donor and cultured in Media 200 (Invitrogen, Paisley, UK) supplemented with low serum growth supplement (LSGS) containing 2% fetal bovine serum, 1 µg/µl hydrocortisone, 10 ng/ml epidermal growth factor, 10 µg/ml heparin, 100 U/ml penicillin and 0.1 mg/ml streptomycin at 37°C in a 95% air/5% CO_2_ humidified atmosphere. Cells were passaged every seven days (5000 cells/cm^2^) with the culture medium changed every two days. Cells were passaged using accutase (Invitrogen, Paisley, UK) and growth rate kinetics calculated using the equation:



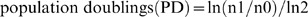
where n0 is the number of the cells seeded and n1 is number of the cells that were counted by countess cell counter (Invitrogen, Paisley, UK) after one week.

Statistical significance of the slopes in the growth curves were calculated using a polynomial second order equation (Y = A+B*X+C*Xˆ2) with the software Graph Pad Prism.

### Assay of senescence-associated -ß-galactosidase (SA-ßgal)

SA-ß-gal activity was determined using a histochemical staining kit according to the manufactureŕs instructions (Sigma-Aldrich). Briefly, HUVEC monolayers were rinsed twice in phosphate buffered saline (PBS), fixed at room temperature for 6–7 of the slopes in the growth curves were calculated min in 2% formaldehyde/0.2% glutaraldehyde, washed three times in PBS and finally incubated at 37°C with SA-ß-gal staining solution (1 mg/ml 5-bromo-4-chloro-3-indolyl ß-D galactoside, Sigma-Aldrich, Sweden) in buffer containing 100 mM citric acid, 200 mM sodium phosphate, 5 mM potassium ferrocyanide, 5 mM potassium ferricyanide, 150 mM NaCl, and 2 of the slopes in the growth curves were calculated mM MgCl_2_ at pH 6.0. The percentage of SA-ß-gal stained HUVECs was determined by counting at least 1,000 cells using light microscopy.

### Reverse Phase Protein Array (RPPA)

#### Protein extraction

The harvested HUVECs from week 1, 6 and 10 were washed twice in ice cold phosphate buffered saline and centrifuged at 4°C, 200 g for 10 min. Protein extraction was performed by dissolving the cell pellet in extraction buffer EXBplus (Qiagen, Hilden Germany) and incubated on wet ice for 15 minutes followed by incubation at 95°C for 10 min. Cells were centrifuged at 4°C, 20,000g for 15 min. Supernatants were collected and stored at −80°C until further analysis. Protein concentrations were determined using the Bradford protein assay (Bio-Rad laboratories, Munich, Germany).

#### RPPA analysis

RPPA analysis was performed as previously described [24]. RPPAs were generated using the SpotBot Extreme Microarray Spotter according to the manufacturer's instructions (Arrayit Corporation, Sunnyvale, CA, USA). Each lysate was arrayed (undiluted, 1∶2, 1∶4, 1∶8, 1∶16 and buffer) in duplicates on to a nitrocellulose-coated glass slide (Oncyte Avid, Grace Bio-Labs, bend, OR). A total of 12 data points were obtained per lysate in order to be able to perform the analysis in the linear dynamic range. The array slides were blocked by using peroxidase blocking solution (DAKO, Denmark) for one hour followed by 5% milk powder in TBS-T for one hour and incubated with the primary antibodies overnight. After washing with TBS-T (3×10 minutes) slides were incubated with appropriate horseradish peroxidase conjugated secondary antibody. Signal detection by chemiluminescence was performed similar to immunoblot analysis. To estimate the amount of total protein, arrays were stained in parallel with Sypro Ruby protein Blot Stain (Molecular Probes, Eugene, USA) according to the manufacturer's instructions. Antibodies had been validated by individual immunoblotting prior to performing RPPA. A complete list of senescence-associated antibodies along with information consisting of the name, type, molecular weight, company name, company number, primary source, and preparation of primary antibody is shown in Table 1.

**Table 1:** List of selected antibodies analyzed using RPPA.

#	Primary antibody (PAb)	Type	1.4 mGy/h week 10	2.4 mGy/h week 10	Mw (kDa)	Company name	Number	Source
1	Akt	Signal transduction	+	Un	60	Cell signalling	#9272	Rabbit
2	Caldesmon	Cytoskeletal protein	Un	–	70–80, 120–150	Cell signalling	#2980	Rabbit
3	Caspase 3	Apoptosis	Un	Un	17, 19, 35	Cell signalling	#9662	Rabbit
4	Desmin	Cytoskeletal protein	Un	Un	53	Cell signalling	#5332	Rabbit
5	Fascin	Cytoskeletal protein	Un	Un	55	Abcam	ab78487	Mouse
6	HIF1-alpha	Transcription factor	Un	Un	120	BD Biosciences	610959	Mouse
7	Hsp 60	Stress responsive proteins	Un	Un	60	Abcam	#ab46798	Rabbit
8	mTOR	Serine/threonine protein kinase	–	–	298	Cell signalling	#4517	Rabbit
9	NF-kB p65	Transcription factor	Un	Un	65	Cell signalling	#3034	Mouse
10	Nucleolin	Nucleolar protein	–	Un	100	EMD Millipore	05–565	Mouse
11	p38 MAPK	Signal transduction	Un	Un	43	Cell signalling	#9212	Rabbit
12	p44/42 MAPK	Signal transduction	Un	Un	42, 44	Cell signalling	#9102	Rabbit
13	Phospho NF-kB p65 (Ser536)	Transcription factor	Un	Un	75	Cell signalling	#3031	Rabbit
14	Phospho PTEN (Ser380)	Tumor suppressor	Un	Un	54	Cell signalling	#9551	Rabbit
15	Phospho-Akt (Ser473)	Signal transduction	–	–	60	Cell signalling	#4060	Rabbit
16	Phospho-HSP27 (Ser 78)	Stress responsive proteins	Un	Un	27	Cell signalling	#2405	Rabbit
17	Phospho-p38 MAPK (Thr108/Tyr182)	Signal transduction	Un	Un	43	Cell signalling	#4631	Rabbit
18	Phospho-p44/42 MAPK (Thr202/Tyr204)	Signal transduction	–	–	42, 44	Cell signalling	#9101	Rabbit
19	Phospho-STAT3 (Tyr705)	Transcription factor	Un	Un	79,86	Cell signalling	#9145	Rabbit
20	Phospho-STAT5 (Tyr694)	Transcription factor	Un	Un	90	Cell signalling	#9358	Rabbit
21	Phospho-VEGFR2	Platelet-derived growth factor	Un	Un	230	Cell signalling	#2478	Rabbit
22	PI3K	Intracellular signal transducer	–	–	85	Cell signalling	#4292	Rabbit
23	PTEN	Tumor suppressor	Un	Un	54	Cell signalling	#9552	Rabbit
24	Ras	Ras family	Un	Un	21	Cell signalling	#3965	Rabbit
25	Rho GDI	Rho GDI pathway	–	–	26	Epitomics	2751–1	Rabbit
26	ROCK	Rho-associated Kinase	Un	Un	160	Cell signalling	#4035	Rabbit
27	SAPK/JNK	Signal transduction	Un	Un	46,54	Cell signalling	#9252	Rabbit
28	SAPK/JNK-Phospho (Thr183/Tyr185)	Signal transduction	Un	Un	46, 54	Cell signalling	#9255	Mouse
29	Serpine (PAI1)	Plasminogen activator inhibitor	Un	Un	45	Serotec	A8P1100	Goat
30	Snail	Transcription factor	Un	Un	37	Selber-gemacht		Rat
31	STAT3	Transcription factor	Un	Un	79,86	Cell signalling	#9132	Rabbit
32	STAT5	Transcription factor	Un	Un	90	Cell signalling	#9359	Rabbit
33	SUMO1	Small ubiquitin-like modifier	Un	Un	90	Santa cruz	Sc5308	Mouse
34	VEGFR-2	Platelet-derived growth factor	Un	Un	210, 230	Cell signalling	55B11	Rabbit
35	Vimentin	Cytoskeletal protein	Un	Un	57	DAKO	M0725	Mouse

The antibodies were chosen to represent senescence-associated proteins. Differentially regulated proteins at week 10 are marked with +  =  up-regulated, −  =  down-regulated or “un”  =  unregulated.

#### Quantification of proteins

To assess protein expression quantitatively TIF files of the Sypro Ruby and antibody stained slides were generated using a Scanjet 3770 gray scale scanner (Hewlett-Packard, Hamburg, Germany). The intensity of data points was quantified utilizing MicroVigene 3.5.0.0 software (Vigenetech, Carlisle, MA). The MicroVigene signal-intensity (MVS) was calculated as the integral of a logistic four-point fit model, which was matched optimally to the 12 data time points that were obtained. The MVS for each antibody was normalized to total protein MVS. Results were evaluated by t-test and a value of p≤0.05 was considered to denote statistical significance. Data are reported as ± SEM.

### Immunoblotting

Proteins were isolated from exposed and control cells after washing with PBS using lysis buffer. Proteins were then separated by SDS-PAGE and transferred to nitrocellulose membranes (GE Healthcare) using a TE 77 semi dry blotting system (GE Healthcare) at 1 mA/cm for 1 h. Nitrocellulose membranes were blocked using 3 % BSA in PBS, pH 7.4, for 1 h at room temperature, washed three times in 10 mM Tris-HCl, pH 7.4, with 150 mM NaCl for 5 min and incubated over night at 4°C with antibodies against p21 (#2947, Rabbit monoclonal, Cell Signaling), p53 (Sc126, Mouse monoclonal, Santa Cruz Biotechnology), and phospho-p53 (Ser-15) (#9286, Mouse monoclonal, Cell Signaling), p16 (Sc-81613, Santa Cruz Biotechnology) using dilutions recommended by the manufacturer. Actin (sc1616, Santa Cruz Biotechnology) was used as a loading control. After washing three times, blots were incubated with the appropriate horseradish peroxidase-conjugated secondary antibodies (Santa Cruz Biotechnology) for 1 h at room temperature and developed using the ECL system (GE Healthcare) following standard procedures. Quantification of immunoblot bands was performed on digitized images using TotalLab TL100 software (www.totallab.com).

### Proteome analysis

#### ICPL labeling of proteins and 1D PAGE separation

**Figure 1:**
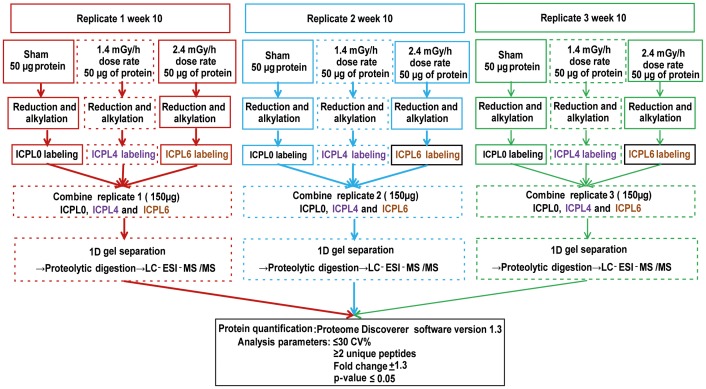
Schematic representation of the ICPL-triplex proteomic approach work flow. The protein samples from week 10 (control and two irradiated) were reduced and alkylated before labeling with ICPL0, ICPl4 and ICPL6. Samples were mixed and further separated using 1D gel electrophoresis and digested as described in Methods. Samples were analyzed by LC-ESI-MS/MS. Quantification of proteins was performed by Proteome Discoverer software using three biological replicates.

Passaged cells at culture week 10 from sham, 1.4 mGy/h and 2.4 mGy/h dose rates were collected for protein analysis using accutase (Invitrogen, Paisley, UK) for detachment. All experiments were performed with three biological replicates. Proteins were isolated using Isotope Coded Protein Labeling (ICPL) lysis buffer (SERVA Electrophoresis GmbH, Germany) and the protein concentration determined by Bradford assay as above. The proteins were extracted form cells cultured for 10 weeks and labeled with ICPL triplex reagent (SERVA) according to the manufacturer's protocol. Schematic representation of labeling and analysis parameters from three replicates is shown in Fig. 1. To control the labeling efficiency, spiked protein (bovine carbonic anhydrase 2) (SERVA) was added to all samples. Triplicate aliquots of 50 µg of proteins obtained from either control or irradiated samples were labeled according to manufacturer's instructions (SERVA). Control and irradiated samples (1.4 mGy/h and 2.4 mGy/h) were labeled using light ICPL0 (L), medium ICPL4 (M) or heavy ICPL6 (H) labeled reagents, respectively. The labeled samples were combined and separated by 12% SDS-polyacrylamide gel electrophoresis [25] before staining by Coomassie blue staining.

#### LC-ESI-MS/MS analysis

After Coomassie blue staining, each lane in the gel was cut into 5 equal slices and subjected to in-gel digestion with trypsin (Sigma Aldrich) as described previously [26]. The digested peptides were concentrated on a nanotrap LC column (300 μm inner diameter ×5 mm, packed with Acclaim PepMap100 C18, 5 μm, 100 Å; LC Packings) before being separated by reversed phase chromatography (PepMap, 25 cm, 75 µm ID, 2 µm/100 Å pore size, LC Packings) operated on a nano-HPLC (Ultimate 3000 RSLC, Dionex) with a nonlinear 170 min gradient using 2% CAN/0.1% FA (A) and 70% CAN/0.1% FA (B) at a flow rate of 300 nl/min. The gradient settings were as follows: 0–140 min: 5–50% B, 141–145 min: 50–95% B, 146–150 min: stay at 95% B and subsequently equilibrate for 20 min to starting conditions.

#### Identification and quantification of proteins

Eluted peptides were analyzed by a LTQ Orbitrap XL mass spectrometer (Thermo Scientific) equipped with a nanospray ionization source, operated in the data-dependent mode to automatically switch between Orbitrap-MS and LTQ-MS/MS acquisition. Full scan MS spectra (from m/z 300 to 1500) were acquired in the Orbitrap with a resolution of 60,000. Up to ten of the most intense ions were selected for fragmentation in the linear ion trap using collision-induced dissociation, depending on signal intensity. High resolution MS scans in the Orbitrap and MS/MS scans in the linear ion trap were acquired in parallel. Target peptides already selected for MS/MS were dynamically excluded for 60 seconds.

The MS/MS spectra were searched against the ENSEMBL Human database (Version: 2.4, 96580 sequences) using the MASCOT search engine (version 2.3.02; Matrix Science) with the following parameters: a precursor mass error tolerance of 10 ppm and a fragment tolerance of 0.6 D. One missed cleavage was allowed. Carbamidomethylation of cysteine was set as a fixed modification. Oxidized methionine and ICPL 0, ICPL-4, and ICPL-6 for lysine were set as variable modifications.

Data processing for protein identification and quantification of ICPL- labeled proteins was performed using Proteome Discoverer version 1.3.0.339 (Thermo Scientific) as described before [23], [27]–[29]. Briefly, Proteome Discoverer software performed automated statistical analysis of the results and used unique peptides to calculate accurate relative protein quantification. The complete peptide and protein profiles were filtered using high peptide confidence and top one peptide rank filters. The MASCOT Percolator algorithm was used for the discrimination between correct and incorrect spectrum identifications [30], with a maximum q value of 0.01. The false discovery rate (FDR) was calculated at the peptide level for all experimental runs using the Decoy option in MASCOT; the significance threshold was set to 0.05. Proteins with a lower score were manually scrutinized and regarded as unequivocally identified if they fulfilled the following four criteria: (a) they had fragmentation spectra with a long, nearly complete y- and/or b-series; (b) all lysines were modified; (c) the numbers of lysines predicted from the mass difference of the labeled pair had to match the number of lysines in the given sequence from the search query and (d) at least one mass of a modified lysine was included in the detected partial fragment series [31]. Due to these criteria, the ICPL method noticeably lowered the significance level of a protein score and increased the probability of a significant protein hit. Differentially-labeled isotopic pairs were detected with a mass precision of 2 ppm and a retention time window of 0.3 min. The calculated peptide ratio variability in the Proteome Discoverer software was used as an alternative of coefficient of variation to calculate a particular protein ratio. For the quantification data as replicates, the software calculated the protein ratios for single searches as a coefficient-of-variation for log-normal distributed data and then calculated a classical coefficient variation for these ratios.

The technical variation between replicates was validated using spiked protein test mixture containing bovine carbonic anhydrase with light to medium to heavy ratio of 1∶1∶2. Significant fold changes were determined by technical variability based on the average values of the CV (10.5%) of spiked protein. Proteins identified by at least 2 unique peptides in two out of three replicates, quantified by the H/L and M/L variability of 2–30% and with a p-value smaller than 0.05 (t-test) were considered for further evaluation. We considered the variability of 30% (>2 CV) as significant as it overcomes technical variability in our experiments. Therefore, proteins with ratios of H/L label greater than 1.3-fold or less than −1.3-fold were defined as significantly differentially expressed. The biological significance of this fold change cut off is in good agreement with the previously published data [23], [32], [33].

#### Network and canonical pathway analysis

Network analysis was performed from the proteomic data with the software tool Ingenuity Pathway Analysis (IPA) (INGENUITY System, http://www.INGENUITY.com) [34] for significant differentially regulated proteins. IPA scores were used to estimate the significance of the network predictions. The randomness of a biological function or network obtained by IPA was determined by calculating the p-value using Fischer's exact test. The score for each network was a numerical value to approximate the degree of relevance and size of a network to the molecules in the given dataset. A score of ≥2 indicated a confidence of 99% and that the network was not generated by a random chance. IPA also provided canonical signaling events by using pathway libraries derived from the scientific literature describing known associations between proteins. The top canonical lists were present as a ratio of number of proteins found in the proteomic analysis divided by the number of total proteins belonging to a certain pathway. Statistical significance (p-value) was calculated by using Fischer's exact test. Differentially expressed proteins at week 10 were analyzed by Gene Ontology (GO) category 'Biological process' using Human ENSEMBL ID's and Protein Analysis through Evolutionary Relationships (PANTHER) database software (http://www.pantherdb.org/).

## Results

### The dose rate of 2.4 mGy/h significantly inhibited growth and induced premature senescence in HUVECs

**Figure 2:**
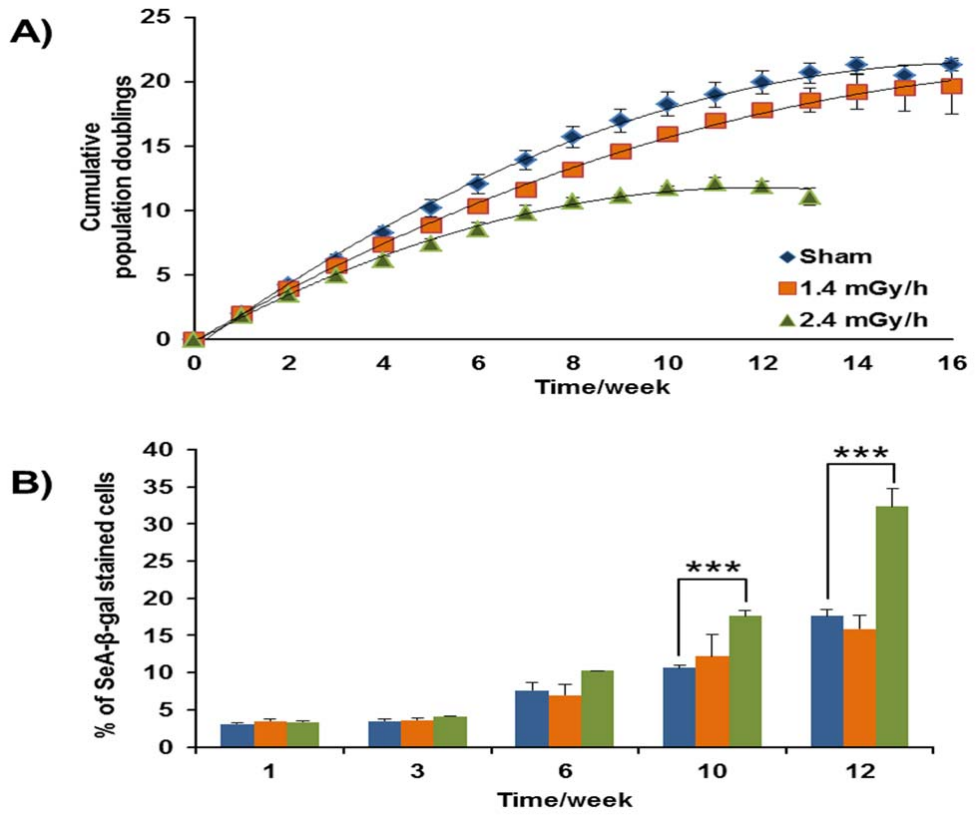
Growth curve and senescence associated β-gal of HUVECs exposed to chronic low-dose rates. A) The growth curve is plotted with cumulative population doublings versus time. Growth curves of control (blue), 1.4 mGy/h (orange) and 2.4 mGy/h (green) irradiated HUVECs are shown. Cumulative population doublings from each week are presented as means ± SEM (n = 3). B) Histograms of positively-stained cells for SA-ß-gal (senescence marker) for control (blue), irradiated by 1.4 mGy/h (orange) and irradiated by 2.4 mGy/h (green). Data are presented as means ± SEM (n = 3). (Students t-test; *p<0.05, **p<0.01 and ***p<0.005).

The number of cumulative population doublings (CPD) was measured for control and ±0.4 CPD (Fig. 2A) and the corresponding value for cells exposed to a dose rate of 2.4 mGy/h was 11±0.6, indicating an overall 50% decrease in the growth rate. For cells chronically exposed to a dose rate of 1.4 mGy/h, a value of 19.6±2.5 was observed, similar to that of control cells.

Statistical analysis with the polynomial second order equation showed no significant change between the slopes of sham irradiated and 1.4 mGy/h curves. Whereas the slope of 2.4 mGy/h curve was significantly different from both sham irradiated and 1.4 mGy/h.

The number of senescent cells was estimated by SA-ß-gal staining at weeks 1, 6, 10 and 12 (Fig. 2B). The number of blue-stained cells was doubled in cells exposed to a higher dose rate exposure in comparison to control cells at week 10, indicating radiation-induced premature senescence. HUVECs exposed to the 1.4 mGy/h dose rate showed no increase in the number of stained cells compared to control.

### Reverse phase protein array and immunoblotting of prematurely senescent cells show association with p21-mediated pathway and altered PI3K/Akt/mTOR and Rho GDI signaling

Analysis of the changes in protein expression accompanying senescence was carried out using cells harvested at weeks 1, 6 and 10. The cumulative radiation doses at these time points were 0.40 Gy, 2.41 Gy and 4.03 Gy for the higher 2.4 mGy/h dose rate, and 0.24 Gy, 1.41 Gy and 2.35 Gy for the lower 1.4 mGy/h dose rate and respectively.

**Figure 3:**
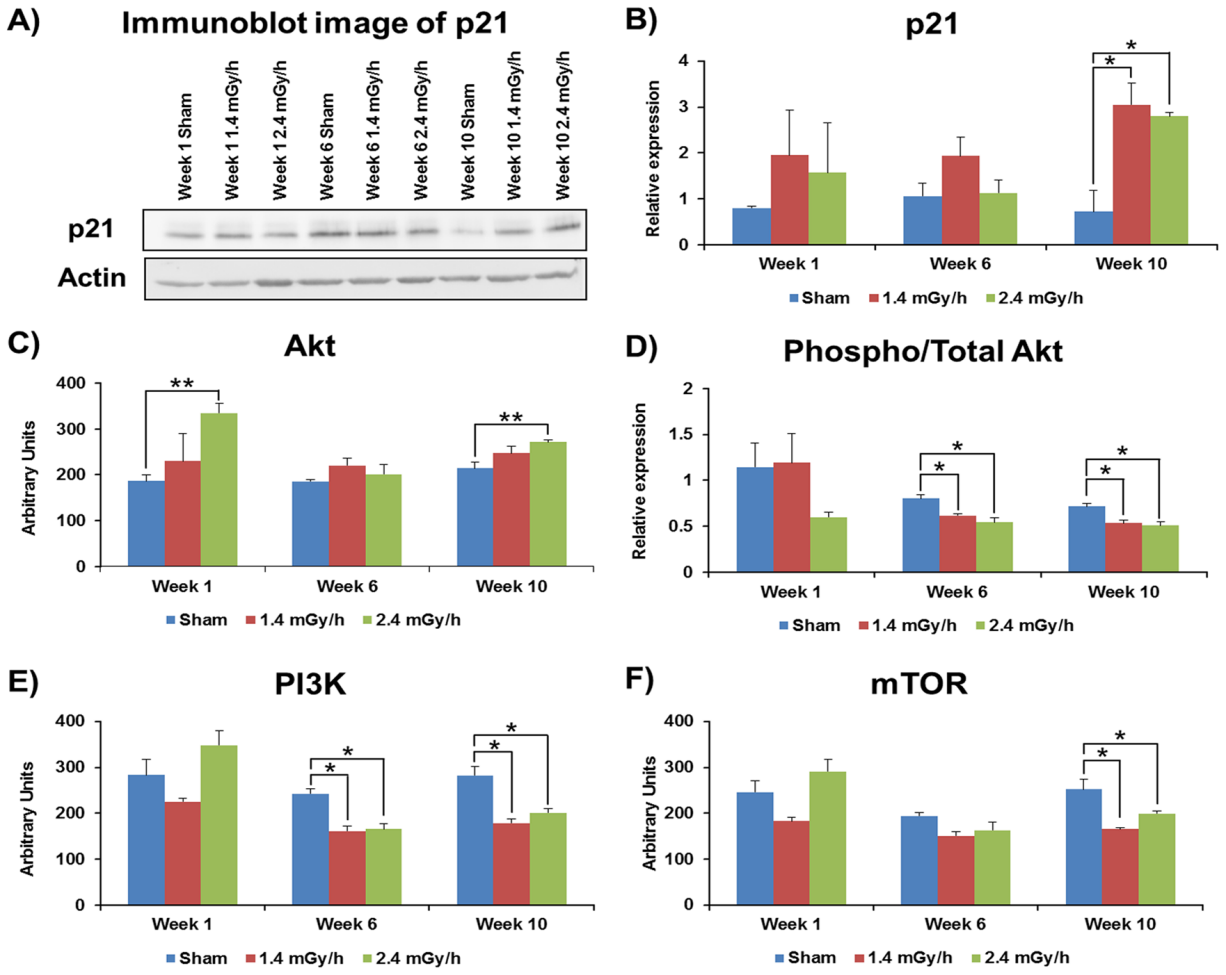
Immunoblot and RPPA analysis of the senescence-associated proteins p21 and members of PI3K/Akt/mTOR pathway. A representative immunoblot of p21 expression levels at different time points and dose rates is shown (A). The columns represent protein levels of p21 (B) Akt (C), phospho-Akt (D), PI3K (E) and mTOR (F) in control (blue), 1.4 mGy/h (orange) and 2.4 mGy/h (green) irradiated HUVECs. The average ratios of relative protein expression in control and irradiated samples are shown. The protein bands were quantified using TotalLab TL100 software by integration of all the pixel values in the band area after background correction and normalized to the actin expression. The data are represented as ± SEM. Three biological replicates were used in all experiments. (Students t-test; *p<0.05, **p<0.01 and ***p<0.005).

Cell cycle inhibitor protein p21, a known marker of senescence [35], was assessed by immunoblotting (Fig. 3A). It was significantly increased at week 10 for both the low and high dose rate exposures (Fig. 3B). No significantly increased protein levels were observed for p53, phospho-p53 or p16 at any time point or dose rate (data not shown).

A panel of senescence-related proteins was analyzed by RPPA; these are listed in Table 1.

As the PI3K/Akt pathway is a known regulator of endothelial senescence [36], [37] we tested involvement of pathway members. The level of total Akt showed a biphasic increase in cells exposed to 2.4 mGy/h observed at early and late time points (Fig. 3C). The ratio of phospho-Akt to total Akt was reduced at both dose rates at weeks 6 and 10 (Fig. 3D). Similarly, the expression of PI3K was decreased at both dose rates in week 6 and 10 samples (Fig. 3E). As Akt is an upstream regulator of mTOR we also measured the level of mTOR expression and found it significantly down-regulated at both dose rates by week 10 (Fig. 3F). Thus, the deregulation of mTOR occurred at a later time point than that of PI3K and Akt.

**Figure 4:**
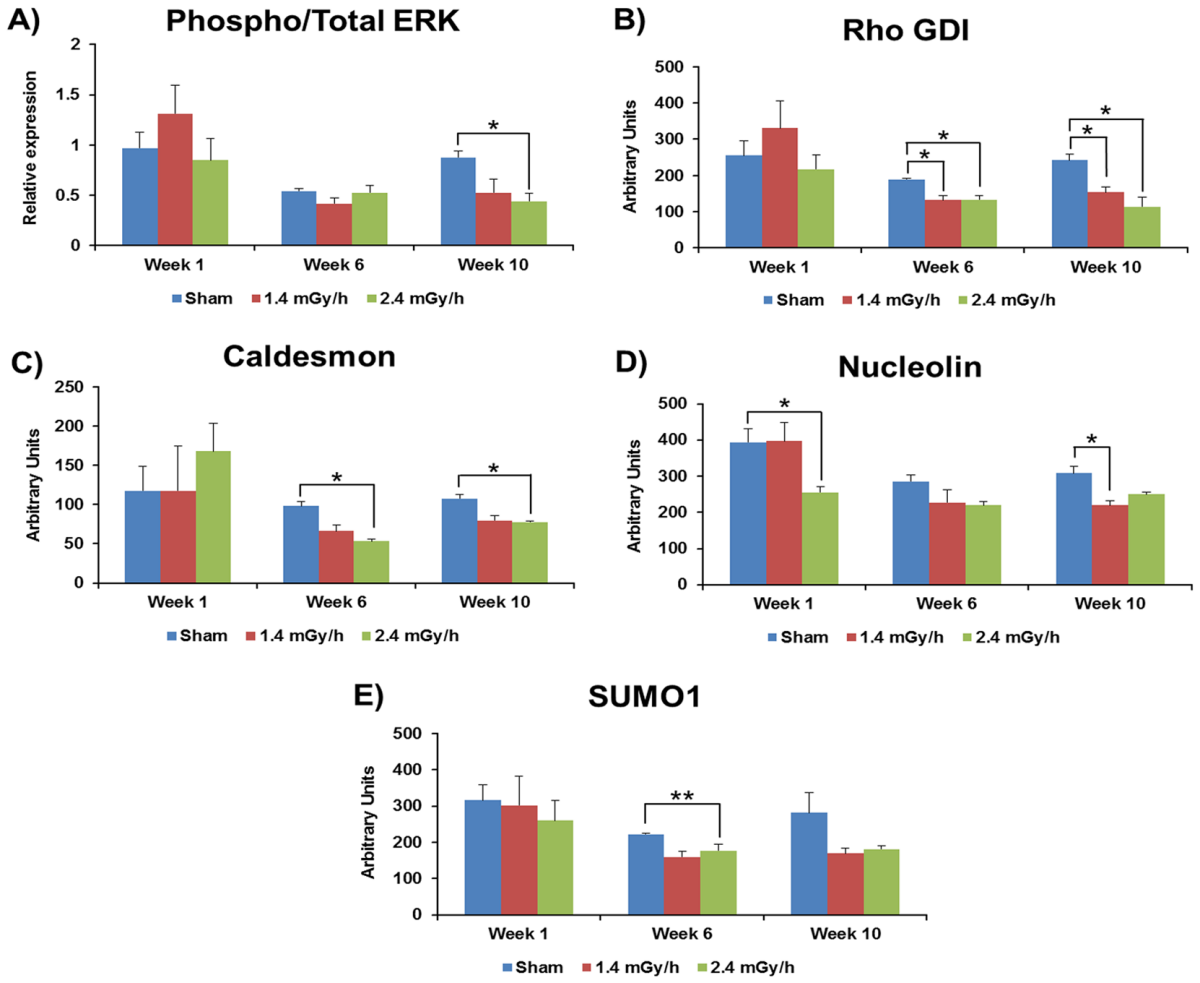
RPPA analysis of senescence-associated proteins. The columns represent protein levels of phospho to total ERK ½ (A), Rho GDI (B), caldesmon (C), nucleolin (D) and SUMO1 (E) in control (blue), 1.4 mGy/h (orange) and 2.4 mGy/h (green) irradiated HUVECs. The average ratios of relative protein expression in control and irradiated samples are shown. The data are represented as ± SEM. Three biological replicates were used in all experiments. (Students t-test; *p<0.05, **p<0.01 and ***p<0.005).

Expression of some proteins shown to be affected at a higher dose rate of 4.1 mGy/h in our previous study [23] were investigated. The ratio of phosphorylated ERK1/2 to total ERK 1/2 was decreased at week 10 at the 2.4 mGy/h dose rate, but not at the lower rate of 1.4 mGy/h (Fig. 4A). Similarly to our previous study [38], expression of Rho GDI that is associated with changes in cellular adhesion and migration, was down-regulated at weeks 6 and 10 at both dose rates (Fig. 4B).

Among other proteins showing differential regulation in the RPPA analysis were caldesmon, showing down-regulation of expression with the 2.4 mGy/h dose rate at weeks 6 and 10 (Fig. 4C), nucleolin that is involved in nuclear transport (Fig. 4D) and the small ubiquitin-like modifier protein (SUMO-1) (Fig. 4E). SUMO-1 is a post-translational modifier protein that showed significant down-regulation with the 2.4 mGy/h dose rate at week 6.

### Proteomic analysis: Canonical pathway and network analysis emphasize the role of mTOR and cell-cell signaling in senescence

Global proteome changes were assessed by ICPL triplex methodology at week 10, the time point showing the most alterations with RPPA. The ICPL triplex labeling of control and irradiated samples followed by LC-ESI-MS/MS analysis identified 2,607 proteins in total. Among 35,546 peptides the number of lysine-containing peptides was 22,587; of these 21,092 peptides were ICPL labeled which corresponds to a labeling efficiency of 93.4%. Quantification of proteins showed that 130 proteins were significantly deregulated at the 1.4 mGy/h dose rate. Of these, 50 were down-regulated and 80 up-regulated (Table S1 in File S1). The number of significantly deregulated proteins identified at the 2.4 mGy/h dose rate was 270 (145 down- and 125 up-regulated) (Table S2 in File S1). There were 68 proteins in common between the two dose rate exposures (Table S1 in File S1). Total identified proteins and peptides are shown in Tables S3 and S4 in File S1, respectively.

The networks where the significant deregulated proteins were involved were analyzed by the IPA software; the biological pathways that were significantly altered by the chronic radiation exposure are shown in Table 2. The pathways of the deregulated proteins at each dose rate were analyzed separately, as was the group of shared proteins.

**Table 2:** Canonical pathways of deregulated proteins.

#	Top canonical pathway	p-value	Ratio
1.4 mGy/h dose rate			
1	EIF2 signaling	8.51E-13	16/174 (0.092)
2	Regulation of EIF4 and p70S6K signaling	1.67E-04	7/151 (0.046)
3	Remodeling of epithelial adherens junctions	2.25E-04	5/65 (0.077)
4	Arginine biosynthesis IV	7.05E-04	2/5 (0.4)
5	Glycogen biosynthesis II (from UDP-D-Glucose)	7.05E-04	2/5 (0.4)

2.4 mGy/h dose rate	p-value	Ratio	
1	EIF2 signaling	6.71E-67	61/174 (0.351)
2	mTOR signaling	6.68E-22	30/185 (0.162)
3	Regulation of EIF4 and p70S6K signaling	1.82E-20	26/151 (0.172)
4	Remodeling of epithelial adherens junctions	1.14E-05	8/65 (0.123)
5	Caveolar-mediated endocytosis signaling	1.56E-04	7/78 (0.09)

Shared pathways between the two dose rates	p-value	Ratio	
1	EIF2 signaling	2.45E-13	13/174 (0.075)
2	Remodeling of epithelial adherens junctions	8.64E-06	5/65 (0.077)
3	mTOR signaling	1.01E-03	5/185 (0.027)
4	Regulation of EIF4 and p70S6K signaling	2.89E-03	4/151 (0.026)
5	Epithelial adherens junction signaling	3.29E-03	4/142 (0.028)

Differentially regulated proteins at week 10 were uploaded to IPA. The most important canonical pathways from both dose rates are shown. Shared pathways between the two dose rates are indicated. Statistical significance (p-value) was calculated using Fischer's exact test. The ratio represents the number of identified deregulated proteins divided by the number of total proteins in that pathway.

The most affected pathway identified at both dose rates was EIF2 (elongation factor 2) signaling, a critical point in regulation of protein synthesis. The number of deregulated proteins involved in this pathway increased with the increasing dose rate (indicated in Table 2, column “ratio”). As a large number of ribosomal proteins were shown to be down-regulated at both dose rates it may be assumed that the rate of protein synthesis was reduced in a general manner in the progression towards senescence.

A second pathway affected mostly at the higher dose rate, but also appearing in the shared pathway analysis (Table 1), was mTOR signaling, confirming the results of the RPPA analysis. EIF4 and p70S6K signaling pathways were down-regulated at both dose rates and consequently also appeared in the shared pathways list. Again, at the higher dose rate many more proteins of this pathway were deregulated. The EIF4 and p70S6K signaling pathway is closely interacting with the mTOR pathway that according to the RPPA analysis was also down-regulated.

The biological functions of the proteins affected by chronic radiation were analyzed by IPA; these, together with the corresponding proteins, are listed in the Table S5 in File S1. The most important biological functions for the lower dose rate were those of cell death and survival, cell cycle and proliferation, whilst at the higher dose rate developmental disorders, protein synthesis and cell morphology were the most affected functions.

**Figure 5:**
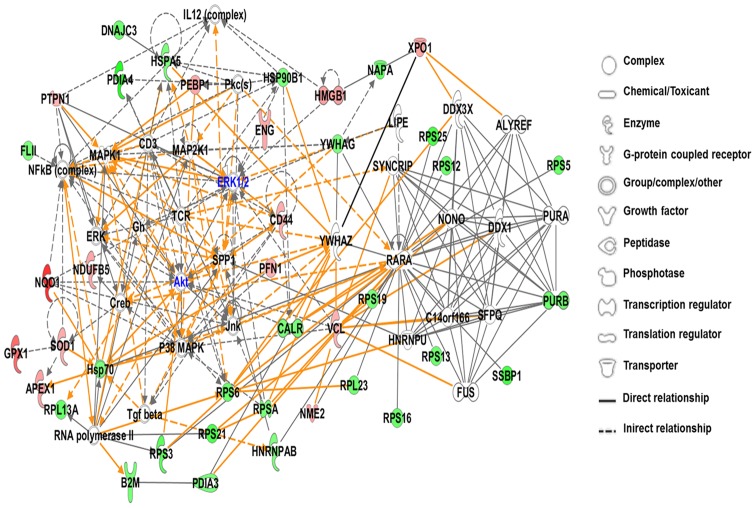
Graphical representation of deregulated proteins representing a merge of two most affected functional networks at week 10 (2.4 mGy/h). Differentially up- or down-regulated proteins are marked in red and green, respectively. Nodal molecules Akt and ERK 1/2 (blue color) were predicted by the IPA software as central transcriptional regulators.

The two networks representing the most important biological functions changed at the 2.4 mGy/h dose rate are shown in Fig. 5. The most important transcriptional regulators of this network were Akt and ERK1/2, indicated in blue.

Significant deregulated proteins (2.4 mGy/h) were also categorized based on their cellular location or metabolic function (Table S6 in File S1). In addition to proteins involved in translational regulation and protein synthesis, a large number of deregulated proteins were constituents of the cytoskeletal structure. Most of these proteins were found to be up-regulated. Other large groups of proteins found to be affected included mitochondrial proteins (10 proteins) and proteins that are involved in the oxidation-reduction homeostasis (10 proteins). The latter showed mostly enhanced expression indicative of oxidative stress. Coupled to oxidative stress are also alterations in proteins involved in the endoplasmic reticulum (ER) (9 proteins). Transmembrane receptors (10 proteins) including endoglin, a member of the TGF beta receptor complex, and two subunits of integrin, were up-regulated.

## Discussion

There is increasing concern about low-dose and low-dose-rate radiation exposure impacts upon human health. It is possible that biological mechanisms responsible for the low-dose-rate effects differ from those of high-dose-rate effects. In radiation-induced CVD, high doses cause overt damage to the myocardium [39] but at low and moderate doses the main target for damage appears to be the endothelium [40]–[42], a cell population typified by a long lifespan, slow proliferation rate and high sensitivity to radiation-induced cell killing. The effects of chronic exposure on endothelial cell function are largely unknown.

We have previously shown that chronic radiation given at the dose rate of 4.1 mGy/h was able to induce premature senescence in primary HUVECs [23]. Several senescence-related biological pathways were shown to be influenced by radiation, including cytoskeletal organization, cell-cell communication, cellular adhesion, and inflammation. Our data suggested that chronic radiation-induced DNA damage and oxidative stress resulted in the induction of the p53/p21 pathway. This is assumed to lead to cell cycle arrest and loss of replicative potential of HUVECs and ultimately to lead to premature senescence.

We have now studied the effects of the lower dose rates of 2.4 and 1.4 mGy/h, and investigated their influence on the senescence status of HUVECs. The higher dose rate (2.4 mGy/h) was able to induce premature senescence based on the appearance of classical hallmarks: the inhibition of growth, and increased number of cells stained by SA-ß-gal. as well as increased expression of the cell cycle inhibitory cyclin-dependent kinase inhibitor p21, a known senescence marker in cellular [43] and animal models [44]. Even at the lower dose rate (1.4 mGy/h), the level of p21 was significantly enhanced in irradiated cells at week 10. However, no effect was seen in SA-ß-gal staining or growth rate. Obviously, the effect of this dose rate is approaching the limit where some of the criteria for senescence are fulfilled but others are not.

A potential signaling pathway involved in the initiation of senescence is indicated by the observed biphasic increase in the level of total Akt (weeks 1 and 10) accompanied by a corresponding decrease of the phosphorylated form (Akt-Ser473) at weeks 6 and 10. In parallel, the levels of PI3K (weeks 6 and 10) and mTOR (week 10) were significantly decreased.

Akt is a mediator of the stimulating effects of vascular endothelial growth factor (VEGF) on endothelial survival and migration [45] where the phosphorylated form of Akt is considered to be biologically active [46]. This activating phosphorylation is regulated by PI3K through the production of inositol phospholipids [47]–[49]. Biochemical and genetic data show that Akt is a positive regulator of mTOR, activating mTOR both directly [50] and indirectly [51]. On the other hand, mTOR regulates the phosphorylation and activation of Akt in endothelial cells [52]. Furthermore, mTOR controls mRNA translation by activating P70S6 kinase and by inactivating the eukaryotic initiation factor 4E (EIF4E) and its binding proteins, which repress mRNA translation [53], [54]. Our proteomic analysis reveals that chronic low-dose-rate radiation (2.4 mGy/h) inhibited the PI3K/Akt/mTOR pathway leading to decreased levels of the downstream targets EIF4E, RPS6, EIF3 and many ribosomal proteins. In contrast, in spite of the decreased levels of PI3K, phospho-Akt and mTOR seen at the lower dose rate of 1.4 mGy/h, the expression of EIF4E or RPS6 was not altered; EIF3 was even slightly but significantly up-regulated. This may explain why the inhibitory effect on cellular growth was not so marked when the lower dose rate was used.

We compared our data to previous studies examining acceleration of senescence and proliferation arrest of endothelial precursors and mature endothelial cells in response to different stimuli [36], [55]–[57]. Mechanisms that were identified in replicative as well as in prematurely-induced senescence included inhibition of PI3K/Akt [36], modulation of cell-cycle regulatory proteins [57], [58], and cell-cycle arrest [58]. Low levels of Akt phosphorylation were associated with stress-induced premature senescence after suppression of PI3K in endothelial cells [59]. Inhibition of PI3K/Akt directly induced premature senescence and increased expression of p21 [60]. Consequently, activation of the PI3K/Akt pathway inhibited endothelial senescence [61], [62]. mTOR activation was shown to promote cell growth and proliferation via activation of ribosomal biogenesis, whereas mTOR inhibition reduced cellular growth [63]. Cellular stress, including DNA damage, was shown to inhibit mTOR signaling [64]. These data are in accordance with the results of this study.

mTOR in its complex 2 form regulates actin polymerization and is thus necessary for actin cytoskeleton formation [65]. Cellular senescence is characterized by disrupted cytoskeletal organization. In the proteomic analysis of prematurely senescent cells exposed to 2.4 mGy/h we found a large number of deregulated proteins (25) belonging to the cytoskeletal structure. The radiation-induced down-regulation of mTOR may lead to alterations in cytoskeletal proteins and contribute to the destabilization of the cytoskeletal structure observed as enlarged and flattened cellular morphology.

Actin cytoskeleton and components of actin-assembly machinery, such as actin-related protein 2/3, are interacting with extracellular matrix receptors such as integrins [66]. In addition, integrin engagement regulates the activity of several members of the Rho family of small GTPases [66]. We found integrin, actin-related protein 2/3 and several members of the Rho family as differentially regulated in this study.

We found a significant down-regulation of Rho GDI with both dose rates at weeks 6 and 10. This down-regulation was, however, not as pronounced as in our previous study when a higher dose rate of 4.1 mGy/h was used [23]. Rho GDI controls the GDP/GTP cycle by suppressing Rho GTPase [67] and regulates the formation of cytoskeleton in HUVECs [68]. Its level was decreased in senescent human dermal microvascular endothelial cells but increased after treatment with anti-aging agents [69]. Interestingly, mTOR has been shown to initiate reorganization of the actin cytoskeleton in a Rho-dependent manner [65].

The Rho pathway, in addition to the cytoskeleton, controls the progression of the G1 phase of cell cycle to the S phase [70]. It has been suggested that the increase of p21 in the senescent cells is due to the action of Rho GDI via the Rho and RAF/MEK/ERK pathway, thus inducing the cell cycle arrest [69]. Our data show significant down-regulation in the level of phospho- to total ERK in HUVECs at week 10 (2.4 mGy/h). Decrease in ERK activity has also been observed in replicative senescence cells [59], [71].

Our study also shows the influence of chronic low-dose-rate radiation on mitochondrial proteins. We have previously shown that, in the case of acute immediate radiation damage, mitochondrial proteins represented the protein class most sensitive to ionizing radiation [29]. Moreover, radiation induced persistent changes in mitochondrial oxidative metabolism and mitochondria-associated cytoskeleton [72], [73]. A significant increase in reactive oxygen species production and protein oxidation was observed in cardiac murine mitochondria four weeks after exposure to a local heart dose of 2 Gy [72]. In line with the previous studies we find here alterations in the expression of proteins involved in oxidation/reduction processes and ER stress indicative of increased oxidative stress. Oxidative stress coupled to mitochondrial dysfunction is considered as a major stimulus for the induction of senescence [74], [75].

## Conclusions

**Figure 6:**
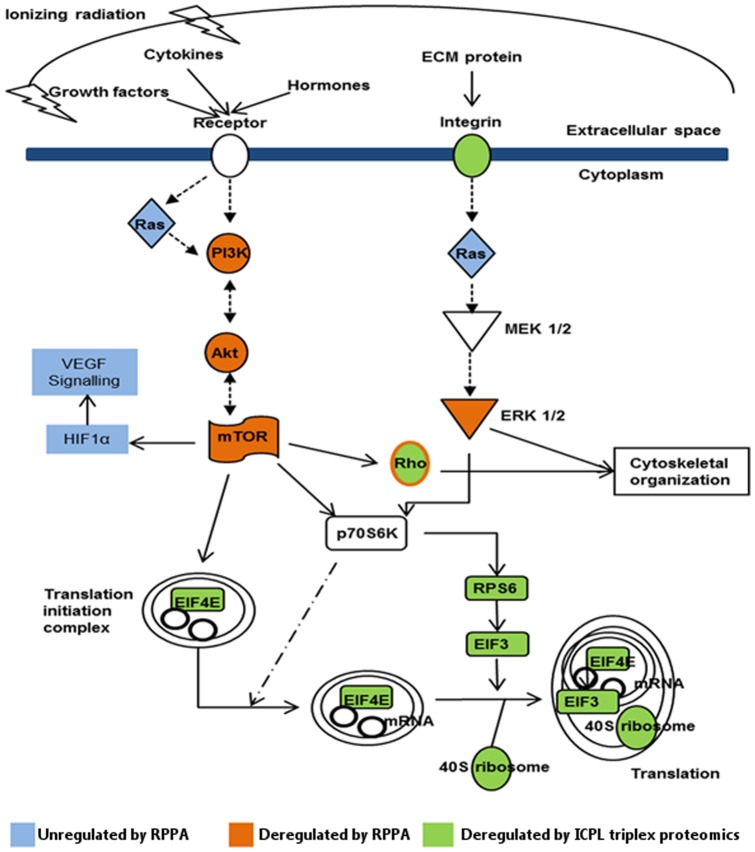
Schematic representation of pathways altered by chronic low-dose-rate radiation in HUVECs. Orange and green colors represent down-regulated proteins detected by RPPA and ICPL triplex proteomic methods, respectively. Blue color indicates unregulated proteins detected by the RPPA technique.

This study highlights the participation of PI3K/Akt/mTOR pathway inhibition in the premature endothelial senescence triggered by chronic low-dose-rate radiation. A schematic presentation of this pathway and its downstream targets is shown in Fig. 6. However, the degree and rate of progressing to senescence is dependent on the dose rate. Consequently, there may be a threshold dose rate under which no effect on endothelial senescence is observed. Although practical significance of this study remains to be confirmed by *in vivo* research, increased understanding of the mechanisms leading to radiation-induced endothelial senescence may provide a basis for preventive measures for CVD seen in populations chronically exposed to low-dose-rate radiation. This work shows the way forward for new target and treatment combinations.

## Supporting Information

File S1 **Table S1, Differentially expressed proteins at week 10, 1.4 mGy/h dose rate.** The proteins listed in the table were identified and quantified by two or more unique peptides. The proteins shared between the two dose rate exposures are indicated in bold. **Table S2, Differentially expressed proteins at week 10 from 2.4 mGy/h dose rate.** The proteins listed in the table were identified and quantified by two or more unique peptides. **Table S3, All identified proteins in this study.** The identified proteins using both dose rates, week 10, are shown. **Table S4, All identified peptides in this study.** The identified peptides using both dose rates, week 10, are shown. **Table S5, Networks obtained from deregulated proteins using Ingenuity Pathway Analysis software.** The networks from 1.4 and 2.4 mGy/h dose rate deregulated proteins are listed. Focus molecules represent the number of deregulated proteins in the network. Names written in bold represent up-regulated and italic down-regulated proteins. Unregulated proteins associated with the corresponding network are written with normal letters. Table S6, Biological functions or cellular locations of deregulated proteins. The proteins deregulated at the dose rate of 2.4 mGy/h, week 10, are shown.
(XLSX)
